# Women’s reasoning and experience in the cervical cancer screening programme when offered a self-sampling HPV test: a qualitative content analysis

**DOI:** 10.1186/s12905-026-04517-9

**Published:** 2026-05-09

**Authors:** Caroline Hellsten, Lina Magnusson, Christer Borgfeldt

**Affiliations:** 1 Department of Obstetrics & Gynaecology, Department of Clinical Science Lund, Skåne University Hospital, Lund University, Lund, SE-221 85 Sweden; 2 Department of Health Sciences, Faculty of Medicine, Lund University, Lund, Sweden; 3 Department of Obstetrics and Gynaecology, Department of Biomedical and Clinical Sciences, Linköping University, Linköping, Sweden

**Keywords:** HPV self-sampling, Acceptability, Experience

## Abstract

**Background:**

Our aim was to explore the reasoning and experiences of women when offered a self-sampling HPV test.

**Methods:**

This study implemented a qualitative study design and content analysis using an inductive approach. Data consisted of written narratives collected through open-ended questions from a total of 173 women. Women were included if they had been offered a self-sampling device since September 2021 and were southern Sweden residents. To achieve purposive sampling with maximum variation, attenders adhering to the screening programme, nonattenders (absent for at least two screening rounds), and women with cervical dysplasia were recruited.

**Results:**

The content analysis generated seven categories: (1) unpleasant experience with a vaginal examination; (2) gratefulness and acceptability of self-sampling; (3) varied perception of one’s capacity to perform self-sampling; (4) preference for cervical sampling by healthcare professionals; (5) anxiety and fear concerning a potential or detected HPV infection; (6) different risk assessments for acquiring an HPV infection; and (7) negative impact on mental well-being due to cervical dysplasia. The overarching theme became “the HPV self-sampling reduced practical and emotional barriers to attending the cervical cancer screening programme, but test results may create anxiety.”

**Conclusions:**

Most women valued HPV self-sampling, although their confidence in performing it varied. Self-sampling can reduce the emotional and practical barriers to participation in cervical cancer screening. However, anxiety about cervical dysplasia or following a positive HPV test was noted, highlighting the need for healthcare professionals to provide personalised information to alleviate negative emotions.

**Supplementary Information:**

The online version contains supplementary material available at 10.1186/s12905-026-04517-9.

## Background

Cytology-based screening, introduced in the late 1960s in Sweden, has traditionally represented the foundational paradigm for cervical cancer screening [1]. However, a growing body of evidence has shown that HPV testing has greater sensitivity than cytology alone [2]. Consequently, primary-based HPV testing now constitutes the cornerstone of cervical cancer screening in many European countries [3], and was implemented in Region Skåne, Sweden in 2017. Vaginal HPV self-sampling can reduce attendance barriers, increase screening coverage and thereby facilitate the detection of early precancerous lesions [4, 5]. In September 2021, HPV self-sampling became the primary screening method in Region Skåne. The recommended screening interval is every five years from 23 to 50 years of age and every seven years thereafter. Women who miss their final screening at age 64 receive annual reminders until age 70.

Women with a positive HPV result from vaginal self-sampling are referred for a follow-up visit, during which a midwife performs cervical sampling for repeat HPV testing and reflex cytology. Those with cytological abnormalities, defined as atypical squamous cells of undetermined significance (ASCUS) or more severe findings, are referred for colposcopic evaluation with biopsy sampling.

When screening protocols, such as the introduction of HPV self-sampling, are updated, it is important to consider women’s preferences and experiences to improve programme quality and increase participation rates. This qualitative study aimed to explore women’s reasoning and experiences when offered an HPV self-sampling test within a cervical cancer screening programme.

## Methods

### Study design

This study was conducted using a qualitative study design, and an inductive content analysis was applied to the data [6]. Content analysis was used to analyse narratives from open-ended questions [7, 8]. An inductive approach was chosen because the study aimed to provide new insights into the acceptability of the self-sampling and explore the reasons for nonattendance [6]. We used the COREQ checklist for reporting qualitative studies [9].

### Sampling strategy and study setting

Women were recruited using purposive sampling [10] and were eligible if they had been offered a self-sampling device in the routine screening programme since its introduction in September 2021 and were residents in Region Skåne. All HPV self-samples and follow-up cervical samples were analysed via the APTIMA mRNA HPV assay (Hologic Gen-Probe, San Diego, CA, USA). To achieve purposive sampling with maximum variation, attenders adhering to the screening programme, nonattenders (absent for at least two screening rounds), and women with cervical dysplasia were deliberately recruited. The variation and distribution in age, ranging from 23 to 70 years, were also considered. The computer programme Flexlabinvitation (Tieto Inc., Stockholm, Sweden) was used to identify attenders and nonattenders of the screening programme on the basis of the inclusion criteria, and women aged 23–29, 30–49, and 50–70 years were randomly selected. All women who were diagnosed with cervical dysplasia and underwent subsequent conization after a positive HPV self-sampling test between 2021 and 2022 were recruited from the Laboratory Information Management System (LIMS) database. To achieve purposive sampling and ensure sufficient recruitment, an invitation letter for study participation was sent to 1,915 women, including 1,500 nonattenders, of whom 750 had collected and returned a self-sample and 750 had not; 300 attenders; and 115 women who underwent conization after a positive HPV self-sample.

### Development and translation of open-ended questions

The questionnaire included nine questions on background information, nine qualitative open-ended questions, and one question asking whether the participant would agree to be contacted with further questions if more information was needed to enrich the dataset (see the Supporting Information, Appendix S1).

The questions were developed by CH and revised by LM, who has experience in qualitative research, and by CB, who has clinical and research experience in the field of cervical cancer [11]. The open-ended questions were tested in June 2022 on three women who had an appointment at the colposcopy unit at Lund University Hospital and four women without cervical dysplasia, which led to minor amendments to the questions. The research team discussed and further revised the questions, resulting in the final version of the questions. The qualitative open-ended questions were translated from Swedish into English by four independent translators whose native language was Swedish but who were fluent in English. Two of the translators were medical doctors, and one had experience in qualitative research. One translation that best reflected the accuracy and content of the original was chosen by CH after discussion with the research team. Back-translation to Swedish was performed by two independent translators and reviewed by the research team. A consensus was reached on the English and Swedish versions of the open-ended questions [12].

### Data collection

In total, 1,915 eligible women were invited to participate in the study via regular mail to their home address; this letter provided information about the study aims and a digital link and QR code to answer the open-ended questions online. The invitations were sent in May 2023, and all the responses submitted before September 31, 2023, were included in the study. Informed written consent was obtained through the first question; only those who agreed could continue. Five women were excluded because they did not provide written informed consent.

### Data analysis

The unit of analysis was the written narratives provided in response to the open-ended questions. The written narratives were read several times to gain a sense of the whole content. The meaning units related to the study aims were then identified, which consisted of words, sentences, or phrases that convey a shared central meaning. These meaning units were then condensed at a descriptive level without altering their original meaning. Finally, a more abstract code was generated for each meaning unit by assigning each meaning unit a word or a sentence to capture its essence. Similar codes were pooled into categories to describe variations in the answers and to express the manifest content [8]. The meaning units, codes, and categories were first outlined by CH, with further revisions and discussions with LM and CB. Ultimately, the emerging categories were analysed and integrated into one overarching theme to illuminate the latent content (Table 1).

**Table 1:** Examples of meaning units, codes and categories

Meaning unit	Codes	Categories
“I think it’s fantastic to have the opportunity to take a sample in such a simple and convenient way, without having to take time off work, for example. It’s much easier for me to use a self-sample than to visit a healthcare facility for the procedure. I can do it at any time of the day that fits into my daily routine.” (24)	Self-sampling is time-efficient and convenient.	Gratefulness and acceptability of self-sampling.
“Receiving a letter stating that I had the infection was a terrible experience. It was deeply upsetting not to have the opportunity to ask all the questions that immediately came to mind. Understanding the nature of the infection—how it is transmitted and its potential consequences—was incredibly difficult. Out of desperation, I turned to the internet for answers, but that often left me feeling even more confused. The delay between submitting the samples and receiving the results was frustratingly long, and the uncertainty significantly impacted my daily life. I became deeply depressed as a result.” (1)	Preferred not to receive information about HPV via letter, which led to searching for information online, and combined with long waiting period for test results, caused feelings of depression.	Anxiety and fear concerning a potential or detected HPV infection.
“I felt mentally overwhelmed during the time I had it (cervical dysplasia). There was little knowledge available, and it’s a topic that no one openly discusses. The entire process was incredibly taxing on my mental health, and to this day, I still feel nervous about the possibility of it (cervical dysplasia) returning. My mental well-being affected my sexual desire and left me regretting having had sex in my life.” (9)	Decreased mental well-being and a diminished sexual desire after being informed about cervical dysplasia and throughout the treatment process, primarily due to lack of knowledge.	Negative impact on mental well-being and sex life due to cervical dysplasia.

To ensure trustworthiness, measures of qualitative criteria for credibility, transferability, dependability, confirmability, and reflexivity were discussed and applied throughout the research process [13]. Strategies used to increase credibility included maximum variation in the purposive sample [10], reaching saturation in data within context, and investigator triangulation [13]. Maximum variation was achieved by purposefully sampling from women who were attenders, nonattenders, or who had cervical dysplasia; who were aged 23–70 years; and who were residing in different cities across Region Skåne. We reached an agreement that data saturation was achieved within each sampling stratum of women. Investigator triangulation was used by the members of the research team, which comprised three researchers with different backgrounds, knowledge, and experiences in the study topic. LM has expertise in qualitative research and public health (associate professor, PhD); CH (PhD student, resident) and CB (professor, MD, PhD) are clinicians with knowledge of the clinical aspects and experience of interactions with women at colposcopy clinics. The neutrality of the data, confirmability, and reflexivity [13] were strengthened by these members of the research team, who contributed to the data analysis from diverse perspectives. The result is transferable to similar contexts. An introduction to the cervical screening programme, and self-sampling approach in Region Skåne were documented and described herein. Consistent documentation of data, considerations, reflections, and decisions that evolved during the research process were maintained and shared as an audit trail to enhance dependability, ensuring the stability of our findings over time [13, 14].

## Results

A total of 173 women contributed narratives in response to the open-ended questions: 101 participated in the previous screening round, 37 were absent, and 45 had a history of cervical dysplasia. The ages ranged from 24 to 71 years, with a mean and median age of 52 years. The characteristics of these women are shown in Table 2.

**Table 2:** Women’s characteristics

	No. of cases (*n*)	%
Level of education		
Primary education	9	5.4
Secondary education	35	20.8
University degree	124	73.8
Native language		
Swedish	140	83.4
Smoking		
Yes	9	5.4
Sometimes	15	9.0
No	142	85.5
Sexual partners		
0–9	109	65.7
10–19	33	19.9
20–29	14	8.4
> 30	10	6.0
Pregnancies		
0	33	19.8
1	18	10.8
2	52	31.1
≥ 3	64	38.3
No contraceptives	116	69
Use of contraceptives	52	31
Condom	18	34.6
Contraceptive cap	1	1.9
Mini pill (progesterone only pill)	4	7.7
Combined oral contraceptive pills	5	9.6
IUD (hormonal coil)	19	36.5
IUD (coil)	5	9.6
Relationship status		
In a heterosexual relationship	111	66.5
In a same-sex relationship	9	5.4
Single and no sexual relationships in the last 6 months	36	21.6
Single with sexual relationships in the last 6 months	11	6.6

*The total sample comprises 173 participants; however, not all participants provided responses to every question. Consequently, the number of observations varies across items, and totals may not sum to 173

**Fig. 1:**
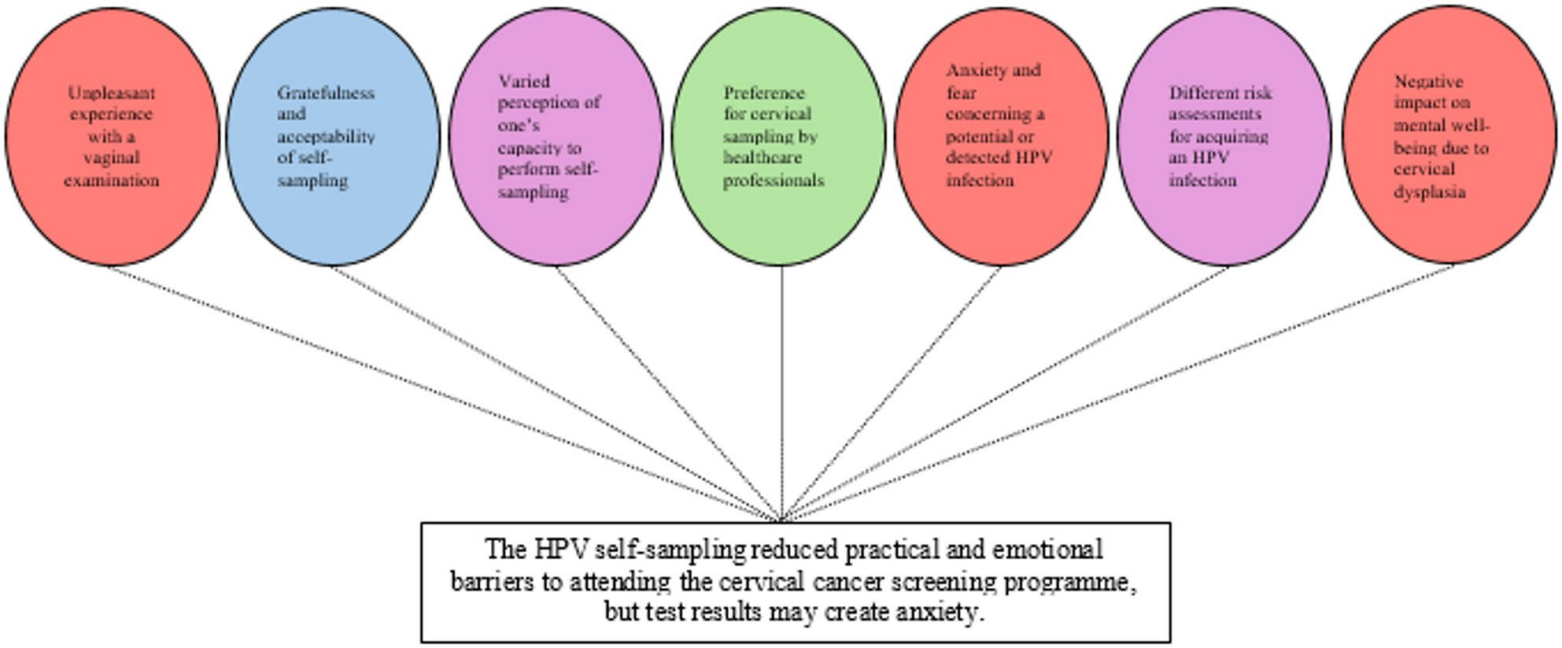
The content analysis resulted in seven categories, which were subsequently merged into one overarching theme

In this qualitative content analysis, seven categories were developed concerning women’s reasoning and experience in the cervical cancer screening programme when offered a self-sampling HPV test. These seven categories were subsequently merged into one overarching theme, as illustrated in Fig. 1.

### The HPV self-sampling reduced practical and emotional barriers to attending the cervical cancer screening programme, but test results may create anxiety

In general, the women appreciated preventive measures and were positive and grateful for self-sampling. A vaginal examination was described as unpleasant despite friendly healthcare professionals. The perceptions of their capacity to perform the self-sampling varied, although most women trusted their own abilities. Some women valued personal contact with healthcare professionals and, therefore, their preference for this method over self-sampling. Few participants reflected on HPV as a sexually transmitted disease. Most women reported anxiety regarding potential or confirmed HPV infections. Overall, cervical dysplasia negatively impacted the mental well-being of those affected, and some women expressed death anxiety, depression, and fear of future infertility. Most women with cervical dysplasia also reported negative effects on their sex life.

The seven categories are presented below, and the numbers in parentheses after the quotations represent the given identification number for each participant.

### Unpleasant experience with a vaginal examination

The vaginal examination was described as unpleasant, painful, too intimate, impersonal, and offensive. It was also described as mentally stressful, uncomfortable, awkward, time-consuming, and filled with shame and vulnerability. Women with pain disorders expressed a lack of understanding from healthcare professionals.

*“I definitely prefer self-sampling. I find gynaecological visits unpleasant and feel exposed*,* therefore I have skipped the pap smears for many years… As I have pain problems*,* the entire visit has been painful and revealing.” (95)*.

Other reasons that contributed to unpleasant experiences included fear of finding other diseases, discomfort in not knowing what procedures were being performed, and the fact that someone else was performing it. Some women described negative experiences regarding professional care due to a lack of empathy, respect, and understanding.

### Gratefulness and acceptability of self-sampling

Trust in healthcare and medical research increased acceptance of self-sampling. The information leaflet was described as pedagogical and comprehensible. The self-sampling was preferred because it was easy and time-efficient, with no need for planning and transportation; it was also reported as more private, convenient, and practical.

*“You can do it yourself; you don’t have to go anywhere*,* you don’t have to take time off from work*,* and the best part – it’s painless. Many midwives are surprisingly rough.” (39)*.

Previous traumatic gynaecological examinations, sexual abuse, and anxiety about health also influenced the preference for self-sampling. The self-sampling was described as less stressful and less painful than a Pap smear. No unnecessary vaginal examinations were performed, and the women expressed their gratitude for the self-sampling.

### Varied perception of one’s capacity to perform self-sampling

Most women trusted their capacity to perform vaginal self-sampling. However, some women expressed uncertainty and anxiety regarding whether the self-sampling was performed correctly; consequently, they found it less reliable. Women with rheumatic diseases, tremors, and obesity found self-sampling difficult. When an HPV infection was detected via the self-sample, its reported reliability increased.

*“I would rather have preferred to do it at the health clinic*,* as it feels safer. It feels uncertain/not as proven to do it at home (with a completely different “brush” as well).” (3)*.

Reasons for doubting one’s capacity to perform the self-sample included uncertainty on how to perform the self-sample according to the instructions. Other concerns involved how the sample should be stored and mailed and the expiration date of the sample.

### Preference for cervical sampling by healthcare professionals

Some women expressed that they valued the personal interaction of a scheduled appointment with a sample performed by a healthcare professional because it provided them with the opportunity to receive guidance with problems regarding reproductive health, undergo a regular checkup, and test for other sexually transmitted diseases.

*“An advantage of having a sample taken by healthcare professionals is that you have the chance to ask questions about possible gynaecological problems. Especially in these times when it is incredibly difficult to get hold of a gynaecologist in Region Skåne.” (118)*.

Another factor that influenced the preference for having a sample performed by a healthcare professional was anxiety after treatment for cervical dysplasia. A personal meeting with a healthcare professional was considered safer and more effortless than a self-sample. The care provided by healthcare personnel was a positive experience and was described as professional, reassuring, empathetic, friendly, informative, and engaging.

Four women expressed an unwillingness to use a self-sampling device. The reasons cited included perceptions that it was inconvenient, unprofessional, and a timesaving and lazy option for the healthcare system. One woman felt affronted to receive the self-sample, whereas another was frustrated by receiving it multiple times without actively choosing it.

Most women expressed that they were pleased with the information on HPV and cervical dysplasia. After the detection of HPV and cervical dysplasia, some women perceived the information as inadequate, which led to increased anxiety, symptoms of depression, and search for answers that were confusing and redundant.

### Anxiety and fear concerning a potential or detected HPV infection

Women expressed feelings of fear, anxiety, sadness, stress, and nervousness about receiving a positive HPV test result. The experience was described as terrifying, and some women reported symptoms of depression. The uncertainty regarding whether the sample could detect HPV infection generated anxiety. Asymptomatic HPV infections and the potential development of cervical cancer are also concerns.

*“Anxiety*,* fear*,* and thoughts about the future… However*,* I had a good doctor*,* and the process that followed felt safe and reassuring.” (36.5)*.

Some women did not consider or worry about the test results. Trust in healthcare professionals and the care they receive, as well as their knowledge that HPV is a common disease, reduces feelings of anxiety and fear.

### Different risk assessments for acquiring an HPV infection

Few women were conscious of the risk of HPV infection, and some had misconceptions regarding the transmission of HPV. The reasons for not reflecting on HPV as a sexually transmitted disease were older age, no sexual contact, or being in a long-term relationship.

*“Never thought about it*,* and if you had gotten it you know where you’ve been. Since I am married and have been for many years*,* I know who to blame.” (21)*.

### Negative impact on mental well-being due to cervical dysplasia

Women expressed feelings of fear, frustration, stress, anxiety, anger, annoyance, discomfort, shock, nervousness, and depression due to cervical dysplasia. The detection of cervical dysplasia also led to anxiety regarding cancer, death, and future fertility.

*“I had high-grade cervical dysplasia but no signs of cancer. However*,* I was afraid that it would lead to the development of cervical cancer. The anxiety was enormous and affected me very negatively… I have started to get used to the thought that I potentially need to defeat cancer and forget my dream to become a mother. My priorities changed. I have been depressed for over 1 year. That’s how long the examination process and treatments went on. Even on the day I got a negative test result*,* I couldn’t stop worrying about my health.” (1)*.

The follow-up period was described as stressful, uncertain, and filled with anxiety; women had a constant fear of reinfection and recurrent cervical dysplasia even after treatment. Decreased mental well-being was attributed to insufficient knowledge, long waiting times, uncertain prognoses, and lack of existing contacts. Some women initially experienced feelings of concern and surprise; however, these feelings were replaced by a sense of safety due to the care provided. The knowledge that cervical dysplasia is common and can be treated at an early stage contributed to the absence of negative effects on mental well-being.

*“My mental well-being is more affected by the tree outside the window that refuses to bloom…” (153)*.

Cervical dysplasia and HPV positivity had an impact on the sex lives of some participants. Women expressed feelings of being “dirty,” “disgusting,” and “limited.” After a positive HPV result, women reported that it was mentally harder to have sex; their sex drive decreased, and for one woman, it disappeared. HPV positivity was considered proof that their partner had been unfaithful. For women who had not been sexually active for a long period of time, a positive HPV result was shocking. HPV positivity and cervical dysplasia also led to a more restrained approach to sex and regret concerning earlier sexual contact.

## Discussion

Overall, the women appreciated preventive measures and were positive and grateful for the HPV self-sampling device. The acceptability of HPV self-sampling for regular attenders of screening programmes has previously been described [15, 16]. For nonattenders, increased participation rates have been observed when they are offered a self-sampling device [5, 17, 18]. Only a few women in this study lacked confidence in their ability to perform self-sampling; the primary reason was their preference for a qualified professional to conduct the sampling. Sultana et al. reported that 81% of women who were underscreened or had never been screened expressed confidence in their ability to perform a self-sampling test correctly. However, 57% of these women reported uncertainty regarding the accuracy of the sample [19]. Confidence in self-sampling and its accuracy is likely to increase as knowledge and acceptance of the device become more widespread. Healthcare professionals and authorities managing screening programmes play a crucial role in educating women about HPV self-sampling, thereby increasing its acceptance.

Preference for the self-sample was attributed to efficiency, convenience, ease of use, and privacy, which is in accordance with two earlier studies that explored women’s experiences with self-sampling [15, 20]. The self-sampling device offers a potential solution that can address practical challenges and improve screening participation rates. We also found a preference for.

self-sampling among women with prior traumatic gynaecological experiences or sexual abuse, allowing them to overcome emotional barriers to attendance.

Vaginal examinations were described as mentally and physically stressful for some women in this study. However, cervical sampling by a healthcare professional was preferred by some women. These women emphasised the value of personal contact and the opportunity to receive a general checkup, where they could ask further questions regarding their reproductive health. Nevertheless, the primary objective of the screening programme is to prevent cervical cancer, not to provide a general gynaecological examination or discussion of health problems.

Anxiety and fear of HPV infection were also reported. Adverse psychological reactions to positive HPV results have been reported in previous studies [21–23]. Maissi et al. reported three variables that predict higher levels of anxiety: younger age, lack of understanding of smear test results, and women who perceive their own risk of developing cancer as being higher [24]. In contrast, long-term anxiety after testing positive for HPV was not observed in two studies [25, 26]. The women in the present study acknowledged the importance of attending follow-up appointments after a positive HPV result. Thus, the initial barrier to participation in a screening programme involving gynaecological examinations may be mitigated following an HPV-positive self-sampling result.

To the best of our knowledge, this is the first study to investigate women’s experiences in a clinical setting where self-sampling is the primary screening method, including both regular attenders and nonattenders. To further increase attendance, studies must be conducted to investigate the acceptance of self-sampling devices and explore suggestions for improvements in the self-sampling approach.

### Strengths and limitations

This study had some limitations. First, the screening test results could not be verified because the responses to the open-ended questions were anonymous. Second, the women may have overestimated or underestimated the time lapse since their last screening test; therefore, the self-reported estimates of their screening programme attendance should be interpreted with caution. Third, women with low literacy may have been underrepresented in the narratives, as most women were well educated. However, the open-ended questions were available in both Swedish and English to minimise the exclusion of non-native Swedish speakers. The proportion of non-native Swedish women in the responding cohort was higher than in the general female population of Region Skåne and among women participating in the national cervical screening programme [27].

A strength of this study was its sampling with maximum variation [10], which included regular attenders, nonattenders, and women with cervical dysplasia in various age groups and cities in Region Skåne. Credibility was further increased by investigator triangulation during the analysis process, which was conducted by three researchers with diverse background knowledge and experience related to the study question [13]. Notably, this study was conducted in a Scandinavian context with publicly funded free cervical screening; nonetheless, the findings should be generalisable to high-income countries with good insurance-based healthcare.

## Conclusion

The results suggest that most women appreciated the self-sample, although their perceptions of their capacity to perform it varied. HPV self-sampling can help overcome the emotional and practical barriers to attending cervical cancer screening programmes. Anxiety related to cervical dysplasia and a positive HPV test were reported; thus, healthcare professionals should provide tailored information regarding individual test results to help mitigate negative emotions concerning test results.

## Supplementary Information

Supplementary Material 1.

## Data Availability

The anonymised data can be made available upon reasonable request to Caroline Hellsten. We have used RedCap (Vanderbilt University, Nashville, TN, USA) for data collection.
