# Supplementary material for: Women’s reasoning and experience in the cervical cancer screening programme when offered a self-sampling HPV test: a qualitative content analysis
Source: BMC Womens Health. 2026 May 9;26:239. doi: 10.1186/s12905-026-04517-9 (PMC13159341; doi:10.1186/s12905-026-04517-9)
Supplement: Supplementary file 1 — Supplementary Material 1. [file 12905_2026_4517_MOESM1_ESM.docx]

I consent to participating in this study

*Yes No*

1. Describe your reason concerning why you chose to (or not to) do a self-test for HPV analysis in the screening programme for cervical cancer?
2. How did you find/experience the information leaflet for the HPV self-test? Is there anything that can be improved?
3. Describe your reason for doing either an HPV self-test or having a test taken by a healthcare professional (e.g., midwife/medical doctor)? *Develop your thoughts further and explain why you prefer one over the other.*
4. Tell us about your previous experience with pap-smear screenings done by healthcare professionals (e.g., midwife/medical doctor)?
5. How do you feel (and explain your thoughts) about the fact that the self-test could possibly demonstrate that you have an HPV-infection?
6. If you have previously had an HPV infection, what can you tell us about your experience?
7. Explain your thoughts around HPV as a sexually transmitted infection (STD)?
8. Please share any other comments about the self-test for HPV, which is part of the screening programme for cervical cancer.
9. If you have or have previously had cellular abnormalities on the cervix, please answer questions a-f, below.
   1. What can you tell us about your experience when you learned that you had cellular abnormalities on the cervix?
   2. How was your mental wellbeing affected?
   3. How was your sex life affected?
   4. How were you met by healthcare professionals?
   5. What was your experience of the information you received regarding cellular abnormalities on the cervix and the HPV virus?
   6. Please share any suggestions for improvements to the healthcare system in light of your experience of learning about cellular abnormalities on your cervix and/or detection of the HPV virus.

**Background information:**

1. How old are you?
2. What is your relationship status?

*Single and no sexual relationships in the last 6 months*

*Single but have had sexual relationships in the last 6 months*

*In a heterosexual relationship*

*In a same-sex relationship*

1. Do you use contraception?

*IUD (coil) IUD (hormonal coil) combined oral contraceptive pills vaginal ring contraceptive patch mini pill (progesteron only pill) contraceptive implant contraceptive injection contraceptive cap condom*

*None*

1. How many times have you been pregnant?

*0 1 2*  ≥ *3*

1. How many sexual partners have you had in your life?

*0-9 10-19 20-29* ≥ *30*

1. Do you smoke?

*Yes sometimes/at parties No*

1. What level of education do you have?

*Primary education Secondary education University degree*

1. What is your native language?
2. Before you received the HPV self-test, when was the last time you participated in the cervical cancer screening programme and had a pap-smear taken?
3. Can we contact you with additional questions if required?

*Yes No*

If you consent to being contacted for additional questions, please supply your phone number and email address below.

Phone number:

Email address:
